# Rapid Symptom Reduction Following Intramuscular Ketamine in Refractory Obsessive–Compulsive Disorder: A Case Report

**DOI:** 10.1155/crps/6450306

**Published:** 2026-08-14

**Authors:** Maxwell Angel

**Affiliations:** ^1^ University of Michigan, Ann Arbor, Michigan, USA, umich.edu

**Keywords:** case report, intramuscular, ketamine, obsessive–compulsive disorder, treatment-refractory

## Abstract

**Introduction:**

Obsessive–compulsive disorder (OCD) is a chronic psychiatric condition that frequently presents with comorbid depressive disorders. For patients exhibiting treatment‐refractory OCD and concurrent depressive disorder(s), there is growing clinical interest in the noncompetitive N‐methyl‐D‐aspartate (NMDA) receptor antagonist ketamine as a rapid‐acting intervention. This case report describes a 27‐year‐old female patient with severe, treatment‐refractory OCD and comorbid depressive symptoms, detailing her response to a single intramuscular (IM) ketamine administration.

**Case Presentation:**

The patient’s primary compulsion was that of handwashing, which she would partake in for ~6 h per handwashing session at the time of the first ketamine administration. This occurred daily over a 3.5‐month period, wherein a single day would consist of up to 18 total hours of handwashing. These extreme compulsions resulted in severe in‐home isolation, precluding in‐person psychotherapy and employment. Despite ongoing management by an independent telepsychiatrist, the patient reported no symptom relief from her prescribed regimen of fluoxetine (80 mg) and alprazolam (1 mg, four times daily)—a pharmacotherapeutic approach that may have been clinically suboptimal given the potential for high‐dose benzodiazepines to exacerbate obsessive–compulsive symptoms. Given the severity of her condition, she subsequently pursued treatment at an unaffiliated outpatient ketamine clinic.

**Conclusion:**

Following a single IM ketamine administration, the patient experienced an acute, ~97% reduction in handwashing duration (from 6 h sessions to 5–10 min sessions) sustained over a 10‐day period. This observation highlights an acute change in the patient’s primary compulsive behavior. However, concurrent pharmacotherapy may confound the results, and prospective controlled trials are required to fully evaluate the efficacy of this intervention.

## 1. Introduction

Obsessive–compulsive disorder (OCD) is a chronic psychiatric condition characterized by the presence of recurrent, troubling obsessions and/or ritualistic, time‐consuming compulsions [1]. Obsessions, as defined by the Diagnostic and Statistical Manual of Mental Disorders, Fifth Edition (DSM‐5), are “persistent thoughts, urges, or images that are experienced as intrusive and unwanted,” causing marked anxiety and stress [1]. Compulsions, often referred to as rituals [2], are defined as “repetitive behaviors or mental acts” performed in an effort to calm or satisfy the anxiety caused by said obsession [1, 3]. The Yale‐Brown Obsessive‐Compulsive Scale (Y‐BOCS), scored from 0 to 40, is the standard clinical instrument for assessing OCD symptom severity [4, 5].

Obsessive–compulsive behaviors, such as cleaning or handwashing, may consume a significant portion of an individual’s day [6]. One such time‐consuming symptom is evident in “contamination fear”—an intense anxiety regarding perceived contamination (incidence rate of 46% [7]). Obsessions of this nature often lead to the compulsive and avoidance behaviors typical of OCD [7].

Often comorbid with OCD (incidence rate of 56.6% [8]) [9], depression is characterized by distressing, impaired mood or anhedonia, present for “most of the day, nearly every day” [10]. The comorbidity of OCD with depression can lead to a worsening of depressive symptoms, particularly with the onset of severe obsessive–compulsive behaviors [9, 11]. This suggests a mutually compounding relationship where improving the primary disorder can positively impact the comorbid condition. Unfortunately, facilitating this relief is generally hindered by the often‐refractory nature of OCD.

OCD treatment resistance is clinically defined as a failure to achieve a 25% or greater reduction in Y‐BOCS scores following adequate therapeutic intervention [12]. Despite the use of selective serotonin reuptake inhibitors (SSRIs) and/or cognitive behavioral therapy (CBT) treatments for OCD, a significant proportion (40%–60%) of patients experience treatment resistance [12, 13]. Similarly, exposure and response prevention (ERP), the leading evidence‐based psychotherapy for OCD, can be challenging to implement [14], particularly for individuals with severe symptoms and/or functional impairment that may preclude effective engagement. In patients displaying such treatment resistance and symptom severity, novel treatment strategies are necessary.

Several glutamatergic methods, including parenteral ketamine and newer oral N‐methyl‐D‐aspartate (NMDA)‐modulating regimens, are currently being explored in refractory OCD to expand therapeutic options. In particular, recent clinical reports have described the use of oral glutamatergic approaches, specifically the Cheung Regimen, which combines dextromethorphan with piracetam, with or without goldenseal root [15]. These novel combinations have demonstrated efficacy in alleviating refractory OCD and related complex psychiatric presentations, representing an emerging therapeutic paradigm prioritizing synaptic plasticity and rapid‐acting NMDA receptor modulation [16, 17].

The psychedelic compound ketamine has emerged as another promising intervention for treatment‐refractory psychiatric conditions, including OCD and depression [18–20]. Its therapeutic effects are primarily attributed to its action as a noncompetitive NMDA receptor antagonist [20–22]. By modulating NMDA‐mediated glutamatergic signaling and promoting neuroplasticity, ketamine is believed to disrupt the rigid neural pathways characteristic of OCD and facilitate novel neural connections [22, 23]. To achieve these therapeutic effects, subanesthetic doses—doses that do not provide deep sedation—of ketamine are administered [24]. Such doses produce a nonordinary state of consciousness in patients, referred to by some researchers as “dissociation” [25].

A feature often described with subanesthetic ketamine is a rapid onset of transient symptom reduction, with relief frequently observed within hours or days [23]. This marked reduction in obsessive–compulsive symptoms promotes a critical therapeutic window. By acutely improving functional capacity and cognitive flexibility, ketamine may facilitate engagement with subsequent psychotherapy (i.e., ERP or CBT) [19, 20, 23], particularly for individuals whose baseline symptom severity previously precluded participation.

However, ketamine therapy for psychiatric disorders lacks standardized FDA‐approved guidelines [26], likely due to a lack of large, randomized controlled studies [27].^1^ Ketamine also presents several common, though transient, adverse effects; these include dizziness, nausea (15%–40% of patients), and temporary increases in blood pressure (10%–50% elevations in blood pressure) and heart rate (HR) [28]. Additionally, risks in long‐term recreational users may include psychological dependence, bladder pathology (such as ulcerative cystitis), and delusions/neuropsychiatric symptoms [28, 29].

The literature is thus divided as to ketamine’s efficacy, with Sharma et al. [30] finding that merely a small “subset of patients with OCD… respond well to ketamine” (11 of 14 reviewed patients experienced no symptom reduction [30]). Conversely, Ceban et al. [28] reviewed that only 1%–5% of patients discontinue treatment due to tolerability and safety concerns. While many private clinics operate under rigorous, provider‐directed internal monitoring protocols,^2^ formal, standardized FDA‐approved guidelines for ketamine administration specifically in OCD currently remain unestablished.

## 2. Case Presentation

### 2.1. Patient History and Baseline

A 27‐year‐old female patient with no known family history of OCD nor a history of psychiatric hospitalization presented to a private outpatient ketamine clinic in the state of Michigan (United States) in early 2025. She and her family reported “extreme”^3^ obsessive–compulsive behaviors, comorbid depression, and symptom perpetuation despite ongoing telepsychiatric treatment. The patient’s symptoms escalated 3.5 months earlier (late 2024) and rapidly evolved from “intrusive thoughts” to debilitating pathogen/cleanliness obsessions and handwashing compulsions. These rendered her incapable of leaving home for ~3.5 months prior to her first intramuscular (IM) ketamine administration.

At the time of the first IM ketamine administration (baseline), the patient exhibited a Y‐BOCS score of 39 (obsessions subscale: 19; compulsions subscale: 20; categorized as “extreme OCD”) and reported severe depression, anhedonia, and anxiety, as well as a loss of ~13.6 kg over the prior 3.5 months. She required assistance in all activities of daily living—including eating, dressing, bathing, and using the restroom—to avoid contamination from perceivedly unclean utensils, electronics, and toiletries. She had also been unable to shower or brush her hair during this period, slept fewer than 4 h each night, was unable to continue employment, and required exhaustive cleaning processes of daily‐use objects. The patient’s cohabitating mother reported that the patient exhibited symptoms consistent with depression since she was a young teenager and that her depressive symptoms worsened with the onset of comorbid OCD.

Though the patient’s obsessions and compulsions are typical of those with OCD [1], it was the duration of the patient’s primary compulsion, handwashing, that truly marked this case as extreme. At the time of the first ketamine administration, a single “handwashing session” lasted up to 6 h, with a single day’s handwashing totaling up to 18 h. The time it took to wash her hands absorbed almost all her waking hours, leading to the aforementioned social and functional impairments.^4^ From excessive cleaning, the patient’s skin was discolored a dark caramel brown from her elbows to her hands. She additionally reported self‐induced fluid restriction to avoid toileting and not necessitate handwashing.

At the time of her presentation, she was prescribed fluoxetine (Prozac; 80 mg, once daily) and alprazolam (Xanax; 1 mg, four times daily) by her telepsychiatrist, who was/is not affiliated with the administering ketamine clinic. While taking both medications, the patient experienced disabling symptom progression, meeting the criteria for treatment‐resistance. At the time of the first administration and during a 10‐day postadministration period, the patient continued taking her medication as prescribed.

While the patient was able to participate in telepsychiatric appointments, these were strictly for medical management and did not include psychotherapy. Both the patient and her mother reported that her isolation precluded engagement in any in‐person psychotherapy, and she thus received none in the time preceding the initial ketamine administration.

### 2.2. The IM Ketamine Administration

The patient received her first IM ketamine administration in early 2025. Upon arrival at the clinic, the patient stated that she was eager to begin, though she had taken 9 h to physically prepare herself for the visit and had carefully donned multiple layers of clothes. Standard physical and neurological exams were conducted with no significant abnormalities other than a thin and anxious appearance, clenched hands/visible tension, and communicated/observed fear of touching objects on her way into the clinic and after sitting down. While IM ketamine administration does not require mandated risk evaluation and mitigation strategies (REMS) like intranasal esketamine [31], continuous monitoring was performed by the attending physician, per the private clinic’s standard practice. The patient refused blood pressure monitoring due to physical touch aversions, though she exhibited normal skin temperature and no signs of poor perfusion or light‐headedness. HR and pulse oximetry (SpO2) were monitored continuously during administration.

At 1:35 PM, an initial dose of 25 mg IM ketamine (~0.5 mg/kg) was administered (vitals: SpO2 99%, HR 73 bpm, RR 20). After 16 min, the patient remained visibly anxious with clenched fists, though reported feeling relaxed. At 1:51 PM, an additional 10 mg IM ketamine was administered for a total approximate dose of 0.7 mg/kg (vitals: SpO2 100%, HR 79 bpm, RR 20). By 2:13 PM, vitals remained stable (SpO2 98%, HR 69 bpm, RR 20). The patient was directly monitored in the room for the entirety of the session and held for a 2 h postinjection observation period.

Shortly after the second dose was administered (2:05 PM), the patient made a request to don the clinic’s headphones, suggesting an acute improvement in her contamination fears. Additionally, she reported further anxiety reduction, stating that she had not “been this calm since being in the womb.” Two hours postinjection and prior to discharge from the clinic, the patient allowed her parents to kiss her on the cheek, a physical interaction they had not had in over 3 months.

### 2.3. Posttreatment Course

The patient reported a degree of continued secondary obsessive–compulsive symptoms, such as the need for the repeated washing of her personal effects and clothes. However, the patient exhibited an immediate and substantial exception to this, seen in her primary symptom. Following the first ketamine administration, the patient’s handwashing duration decreased markedly from ~6 h per posttoileting handwashing session at baseline to 5–10 min per session posttreatment. She also reported that she ceased looking back at the toilet seat following toileting to see whether her shirt had touched the seat. She had done this repeatedly after each episode of toileting for the 3.5‐month period prior to her first ketamine administration.

A formal posttreatment Y‐BOCS was not collected immediately following the first administration. Upon the conclusion of the administration, the patient and her parents shared a profoundly emotional and intimate moment of physical contact. Interrupting this clinical breakthrough to administer a standardized questionnaire was deemed therapeutically inappropriate by the attending physician. Because a posttreatment Y‐BOCS score was not obtained, the overall OCD symptom severity and multidimensional improvement cannot be quantitatively assessed or confirmed. Instead, symptom tracking relied on detailed, corroborated reports from both the patient and her parents. These reports confirmed that the reduction in handwashing to 5–10 min per session was sustained during the 10‐day period leading up to her second scheduled ketamine administration.

Beyond this period, the patient opted to continue ketamine treatment and has exhibited further symptom alleviation. Following her second administration (10 days after the first), she received ketamine administration every 3–5 days. Most notably, following a total of ~3.5 weeks of ketamine treatment, the patient began engaging in ERP with a psychotherapist who was/is not affiliated with the administering ketamine clinic. Her handwashing duration decrease was sustained throughout this time.

## 3. Discussion

The reduction in handwashing duration represents a significant observational change in the patient’s primary compulsive behavior. Approximately 18 h of handwashing precluded the patient’s functionality and induced social isolation, a lack of sleep, and increased anxiety. Handwashing time reduction to 5–10 min per session represents an approximate 97%^5^ reduction in handwashing duration.

This reduction in handwashing time provided the patient with more time in her day for other tasks, correlating with a self‐reported decrease in anxiety and anhedonia. The patient and her cohabitating family reported significant improvement in her quality of life, as well as hope for continued reduction of the patient’s symptoms. The patient also exhibited a marked reduction in anxiety and depressive symptoms, voicing feelings of newfound calmness repeatedly throughout the session and later reporting continued improvements in her symptoms throughout the 10 days leading up to her second session (corroborated by family members). Despite these improvements, the patient did not immediately begin attending psychotherapy sessions due to continued social isolation. She did, as previously noted, begin ERP ~3.5 weeks after the initial administration.

**Figure 1:**
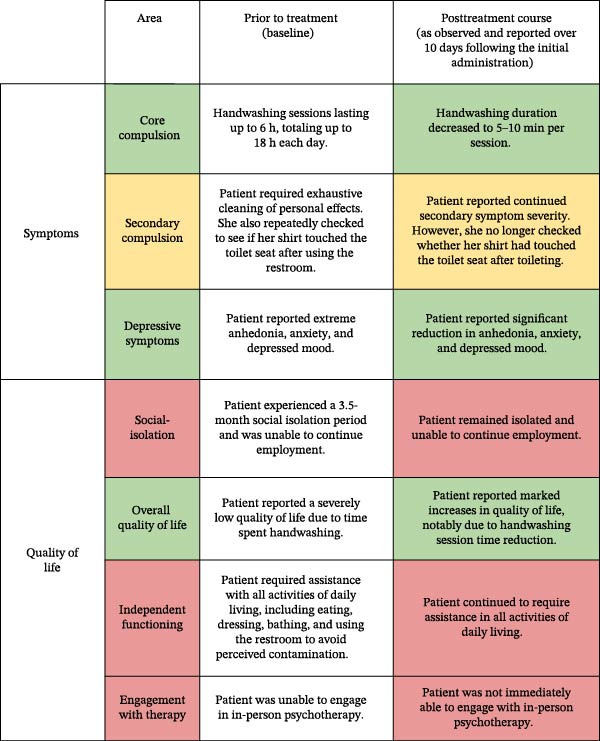
Treatment course diagram.

**Figure 2:**
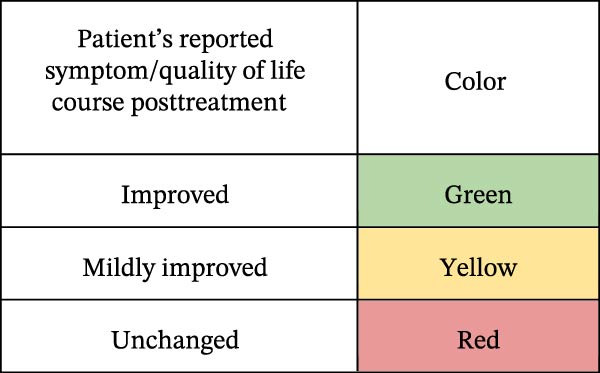
Color key for treatment course diagram.

Thus, the patient experienced alleviation in two symptom areas (see Figure 1, “core compulsion” and “depressive symptoms”) and in one quality of life area (see Figure 1, “overall quality of life”). While there are seven areas listed in Figure 1, these three areas have provided the patient with a profound, novel, reported calmness/decrease in anxiety and a positive outlook on the course of her treatment (Figures 1 and 2).

It should be noted that Figure 1 serves as a qualitative summation of the clinical information discussed throughout this report and is designed to provide a visual overview of the patient’s course. The specific symptom and quality of life areas delineated within the figure were not derived from a formal standardized assessment tool and were instead categorized based on the attending physician’s clinical judgment and patient and family reporting.

Despite these improvements, the patient’s concomitant medication regimen complicates the clinical results and confounds the strict definition of her condition as exclusively “treatment‐resistant” prior to ketamine therapy. While the patient was prescribed 80 mg of fluoxetine (which is typically considered the standard maximum FDA‐approved dosage for OCD), evidence suggests that highly refractory cases sometimes necessitate supratherapeutic dosing to achieve symptom remission [32]. However, her concurrent maintenance on high‐dose alprazolam (1 mg, four times daily) represents a major pharmacological confound throughout the observation. Benzodiazepines function as gamma‐aminobutyric acid receptor (GABA‐A) agonists, enhancing inhibitory neurotransmission and dampening overall neural excitability. This mechanism directly opposes the hypothesized therapeutic pathway of ketamine, which acts as an NMDA receptor antagonist, blockading receptors on GABAergic interneurons to disinhibit pyramidal neurons. This process triggers a transient surge of glutamate and upregulates neuroplastic markers like BDNF and mTOR.

By augmenting GABAergic inhibition, high‐dose benzodiazepines can blunt this glutamatergic surge and attenuate downstream synaptogenesis [33]. Indeed, various studies have demonstrated that concurrent benzodiazepine use can significantly delay, shorten, or dampen ketamine’s rapid antidepressant and neuroplastic effects [33–35]. Psychologically, benzodiazepines are known to potentially inhibit the cognitive habituation and extinction learning processes necessary for successful OCD management and can paradoxically worsen symptoms in some individuals [36], meaning that it is possible that the patient’s preadministration symptomology was exacerbated by the use of alprazolam.

While the patient met the working definition of treatment‐resistant due to her symptom progression, her outpatient pharmacotherapy may not have been fully optimized prior to her presentation at the ketamine clinic. Nonetheless, the patient unusually exhibited rapid and sustained symptom alleviation postadministration. While her behavioral improvement suggests a robust response despite benzodiazepine use, it is difficult to isolate the exact neurobiological mechanism of the patient’s treatment response.

Another clinically notable aspect of this case was the use of IM ketamine as opposed to intravenous (IV) administration. In contrast to IV setups, IM administration bypasses complex IV line preparation, which may ease administration in patients with severe venipuncture‐induced anxiety or contamination fear. However, IM boluses lack the safety benefit of real‐time titration, yielding rapid absorption (peak concentration within 5–30 min; ~93% bioavailability) [37]. While both routes can cause transient cardiovascular elevations requiring vital sign monitoring [38], managing acute adverse effects can theoretically be more complex without an established IV catheter for immediate rescue medication, though low‐dose pediatric data support the general safety profile of the IM route [39]. Compared to established IV ketamine trials in OCD—where single infusions led to a clinical Y‐BOCS response in 50% of a drug‐free cohort at 1 week [20] or yielded a minor, nonresponsive mean Y‐BOCS reduction of only ~ 11% in treatment‐refractory patients at 3‐days postadministration [40]—the present patient’s robust handwashing reduction was atypically sustained over 10 days.

## 4. Discussion of Limitations and Future Directions

A primary limitation of this case is the reliance on subjective reporting and the absence of a formal posttreatment Y‐BOCS. Without psychometric re‐evaluation, overall OCD improvement cannot be quantified, nor can a reduction in core obsessive thoughts or secondary compulsions be formally verified. To fully address this methodological limitation, it is crucial to thoroughly explain why the posttreatment Y‐BOCS was deferred and how the observed 10‐day durability supports a therapeutic transition. As previously delineated, the decision to omit a formal Y‐BOCS directly after the session was based on the attending physician’s clinical judgement. The patient and her parents were engaged in a highly emotional, therapeutic breakthrough characterized by physical contact after 3.5 months of physical isolation. Introducing a clinical questionnaire was deemed countertherapeutic. A follow‐up Y‐BOCS was not remotely administered prior to the second administration due to the patient’s inability to utilize electronics (as a result of her contamination fear^6^). Y‐BOCS administration at the second administration was, once more, deferred to not induce undue anxiety. Additionally, it should be noted that this case was observed at a private clinical practice rather than at a formal research institution.

Without posttreatment Y‐BOCS scoring, it is impossible to determine whether the reduction in handwashing time reflected a broad reduction in OCD severity, a localized reduction in a single compulsive behavior, or an acute postsedative anxiolytic response. However, the acute sedative and dissociative effects of subanesthetic ketamine typically dissipate within 24 h [41]. In this case, behavioral observations confirmed that the reduction in handwashing duration was sustained over the entire 10‐day period preceding her second administration. This subjective data was corroborated by the attending physician and the patient’s parents at the second administration, suggesting that the behavioral response significantly outlasted the acute postsedative window.

While the clinical reports were indeed corroborated, they may contain a degree of inaccuracy due to recall and reporting biases. Expectancy effects may have also biased the patient’s reporting. To minimize recall bias, account for expectancy effects, and improve the strength of clinical claims in future complex cases, researchers should incorporate low‐burden objective symptom tracking. Recommended strategies include daily timed handwashing logs maintained by the patient or cohabitating family, brief daily scales for obsessional and compulsive urge intensity, and/or a delayed Y‐BOCS assessment administered remotely at 24–48 h posttreatment.

The patient’s concomitant medication regimen must also be considered a limitation. High‐dose benzodiazepines may ultimately limit the durability of the “therapeutic window” opened by ketamine’s neuroplastic effects. For clinicians managing complex patients with polypharmacy, a gradual clinically supervised benzodiazepine taper or dose reduction should be considered prior to initiating subanesthetic ketamine whenever feasible. Optimizing the patient’s pharmacological baseline in this manner would likely maximize the postadministration neuroplastic window, prevent the blunting of glutamatergic synaptogenesis, and facilitate more effective engagement with subsequent psychotherapies.

This case report has not fully discussed the patient’s subsequent IM ketamine administrations. While these subsequent administrations have provided further symptom relief, they do not present as marked of a change in symptom severity as the first administration (hence their lack of thorough inclusion in this report). Additionally, because the patient returned to the clinic for a second administration 10 days following the initial administration, it remains unknown exactly how long the effects from the initial administration would have ultimately lasted. This durability is not confounded by psychotherapy as the patient did not begin CBT or ERP until ~3.5 weeks following her initial administration.

## 5. Conclusion

The acute reduction in handwashing duration observed following IM ketamine administration aligns with prior case reports describing acute behavioral changes, though definitive conclusions regarding treatment efficacy cannot be drawn from an uncontrolled, single‐case observation. Although the patient’s social isolation persisted postadministration, the reduction in her primary compulsion correlated with reported decreases in anxiety and anhedonia, as well as improvements in subjective quality of life and treatment outlook. While large‐scale, randomized controlled studies are necessary to establish standardized guidelines and evaluate therapeutic efficacy, case reports such as this and others [42–44] offer preliminary insights into ketamine’s potential to temporarily alleviate severe, treatment‐refractory compulsions.

## Acknowledgments

The author thanks Dr. Abby Bonato for her mentorship and commitment to the editing of this report.

## Funding

The author declares that there was no source of funding for this research. The author was a clinical volunteer.

## Ethics Statement

As this is a retrospective description of a single clinical case, formal Institutional Review Board (IRB) review was not required, as the activity does not meet the federal definition of human subjects research. All clinical care and procedures were conducted in accordance with the ethical principles of the Declaration of Helsinki. While personally identifying information has been removed or generalized, the patient provided explicit, informed written consent for the publication of this case report and the disclosure of unique clinical characteristics in accordance with HIPAA authorization standards. The patient’s informed written consent has been received and archived.

## Conflicts of Interest

The author declares no conflicts of interest.

## Endnotes

1

2

3

4

5

6

## Data Availability

Data sharing is not applicable to this article, as no datasets were generated or analyzed during the current study.

## Footnotes

1
^1^Intranasal esketamine was recently approved by the FDA for use in treatment‐resistant depression [24].

2
^2^As practiced by the outpatient ketamine clinic discussed in this report.

3
^3^See patient’s Yale‐Brown Obsessive‐Compulsive Scale baseline score of 39.

4
^4^Thus, the handwashing resulted in the previously mentioned lack of sleep, unemployment, and need for a caretaker.

5
^5^Calculated from 6 h handwashing sessions being reduced to 5–10 min.

6
^6^The patient’s cohabitating parents may have been able to complete the Y‐BOCS on the patient’s behalf. This, however, may have biased the response to some degree and was again deferred.
